# Integrating Patient‐Reported Quality Measures in Systemic Lupus Erythematosus: Development of the American College of Rheumatology Implementation Guide

**DOI:** 10.1002/acr.80038

**Published:** 2026-04-29

**Authors:** Catherine Nasrallah, Cherish Wilson, Christine Hariz, Christie M. Bartels, Shanthini Kasturi, Wambui Machua, Amy Bennett, Kate Chiseri, Allison Plitman, Starla Hairston Blanks, April Jorge, Patti Katz, Jinoos Yazdany, Shivani Garg

**Affiliations:** ^1^ University of California San Francisco; ^2^ University of Wisconsin School of Medicine and Public Health Madison; ^3^ Tufts Medical Center Boston Massachusetts; ^4^ Piedmont Atlanta Hospital Atlanta Georgia; ^5^ America Heart Association Dallas Texas; ^6^ American College of Rheumatology Atlanta Georgia; ^7^ Allergy and Immunology Unit Massachusetts General Hospital Boston Massachusetts; ^8^ Harvard Medical School Boston Massachusetts; ^9^ Yale University New Haven Connecticut

## Abstract

**Objective:**

To support high‐quality, patient‐centered care for systemic lupus erythematosus (SLE), the American College of Rheumatology (ACR) developed evidence‐based measures incorporating clinical and patient‐reported outcome measures (PROMs). Using the Consolidated Framework for Implementation Research (CFIR), we conducted semistructured interviews with rheumatologists and support staff who piloted these measures. Insights from these interviews informed development of the SLE Quality Measures Implementation Guide for national dissemination.

**Methods:**

Five rheumatology practices were selected to pilot workflows for collecting SLE PROMs recommended in recent ACR guidelines for at least 50 consecutive patients. Measures included physical function (PF) and depression. After data collection, representatives from each practice participated in virtual semistructured interviews. Interviews were recorded, transcribed verbatim, and analyzed thematically using both deductive and inductive techniques. Findings were used to develop the SLE Quality Measures Implementation Guide.

**Results:**

A total of 31 clinicians across five practices participated in the pilot. Interviews with eight participants identified key themes related to innovations, resources, best practices, and challenges in collecting and using SLE PROMs, mapped to CFIR domains. Despite barriers related to workflow, engagement, and resources, PROM implementation in SLE care was well accepted and supported standardized assessment of mental health and PF through communication and electronic health records integration. These insights informed development of the SLE Quality Measures Implementation Guide, featuring guidelines, workflow recommendations, best practices, and educational resources to improve workflow efficiency and enhance patient care.

**Conclusion:**

This study evaluated the implementation of ACR‐recommended SLE PROMs and provides actionable recommendations and resources to address challenges through the SLE Quality Measures Implementation Guide.

## INTRODUCTION

Systemic lupus erythematosus (SLE) is a multifaceted autoimmune disease that challenges both patients and clinicians due to its multisystem involvement, variability, and fluctuating course.^1^, ^2^ The heterogeneity of clinical presentations and the difficulty of diagnosing SLE pose significant challenges in treatment, emphasizing the need for individualized care and consistent monitoring.^1^ In addition, managing SLE requires addressing not only the physical manifestations of the disease but also its significant impact on emotional health.^3^, ^4^ This complexity underscores the need to define how to implement coordinated, multidisciplinary, comprehensive care to improve patient outcomes and support overall well‐being.^5^, ^6^

**SIGNIFICANCE & INNOVATIONS**

- This is the first study to examine the implementation of American College of Rheumatology (ACR)‐recommended patient‐reported outcome measures (PROMs) in systemic lupus erythematosus (SLE) clinical care and to identify barriers and facilitators to collect these measures in routine practice.
- We used the Consolidated Framework for Implementation Research to guide and analyze qualitative semistructured interviews and to examine the successes and challenges of implementing PROMs in SLE clinical care.
- Using a consensus‐building approach, we combined iterative expert discussions, review of current literature, and feedback from semistructured interviews with trial practices that implemented ACR‐developed SLE PROMs to develop the SLE Quality Measures Implementation Guide (http://slequalitymeasures.kotobee.com).
- This guide is the first open‐source, evidence‐based resource to support rheumatologists and care teams in integrating PROMs into routine practice by providing guidance and practical strategies for collecting these measures to inform clinical decisions and advance patient‐centered care.

In recent years, there has been increasing recognition of the importance of incorporating and consistently tracking patient‐reported outcome measures (PROMs) in the routine care of SLE to improve disease management and support patient‐centered care.^2^, ^7^, ^8^, ^9^, ^10^ PROMs provide valuable insights into key aspects of patients’ perception of how their health is affected by SLE that routine clinical assessments may overlook, allowing standardized and validated assessments of symptoms such as fatigue, pain, emotional well‐being, and cognitive function.^7^, ^11^ These dimensions are particularly important in SLE, for which studies have demonstrated that individuals often experience significantly poorer health‐related quality of life compared to those with other chronic conditions.^12^, ^13^, ^14^, ^15^ Furthermore, studies have shown that the rates of depression and anxiety are up to twice as high in patients with SLE compared to the general population.^16^, ^17^, ^18^, ^19^, ^20^ This increased mental health burden, along with the frequent discordance between clinician assessments and patient experiences highlighted in the literature, emphasizes the need for standardized tools such as PROMs to facilitate shared decision‐making and ensure that care is aligned with patient priorities and values.^21^, ^22^, ^23^

To support high‐quality, patient‐centered care, the American College of Rheumatology (ACR) developed evidence‐based longitudinal and quality measures that incorporate the use of PROMs, along with key clinical measures, in the management of SLE.^9^ Although the development of these measures represents a significant step toward more individualized care, the routine use of PROMs in clinical practice remains limited.^7^ To address these gaps and build on the initial phase of this work, which focused on the development of the ACR SLE quality measures,^9^ this study represents part II of the project aimed to (1) investigate the successes and challenges of implementing PROMs for SLE clinical care, and (2) synthesize key implementation findings and tools to inform the development of the SLE Quality Measures Implementation Guide for national dissemination.

## METHODS

### Selection of practices and data collection

Using purposive sampling, five rheumatology practices participating in the Rheumatology Informatics System for Effectiveness (RISE) registry and using the NextGen electronic health record (EHR) system were selected from approximately 100 NextGen RISE practicesfor a pilot study. Practices were selected to represent diverse geographic regions across the United States. Each practice was tasked with implementing workflows to collect SLE PROMs recommended in recent ACR guidelines for at least 50 consecutive patients, including measures of physical function (PF; Routine Assessment of Patient Index Data 3 [RAPID‐3] or Multidimensional Health Assessment Questionnaire [MDHAQ]) and depression assessment (Patient Health Questionnaire 8 [PHQ‐8]).^9^ Practices received support from a NextGen consultant to create structured EHR fields for PROM collection. Data collection for at least 50 PROMs per domain occurred between June 2023 and June 2024.

After data collection was complete, all clinicians (including rheumatologists and medical support staff), as well as practice managers and administrators from the five participating practices, were invited to participate in virtual interviews. Researchers trained in qualitative data collection (CMB, AJ, and JY) conducted interviews via Zoom between August and October 2024. One researcher conducted each interview while other team members took notes. Interviews were guided by a semistructured interview guide based on the domains of the Consolidated Framework for Implementation Research (CFIR) model to explore perceptions about the pilot study and identify barriers and facilitators to SLE PROM implementation. Questions were mapped to CFIR domains and addressed (1) workflows for SLE PROM collection, (2) challenges and opportunities in collecting the measures, (3) practice resources and characteristics, and (4) opportunities to improve current PROM collection and use (Supplementary Material 1). The interview guide was pilot tested and iteratively refined by the research team, the ACR, and collaborating rheumatologists. The study was approved by the University of California, San Francisco, Institutional Review Board. Interviews lasted approximately one hour, were audio recorded, and were transcribed verbatim with transcripts checked for accuracy.

### Conceptual framework

Our study was guided by the CFIR model, which offered a comprehensive structure for examining the factors that affect the implementation and collection of SLE outcome measures in routine care. Widely applied in quality improvement and health research,^24^, ^25^, ^26^ CFIR enables systematic analysis of implementation efforts across multiple levels, helping to describe workflows that overcome challenges and center on opportunities that support adoption and effectiveness of interventions.^27^ This framework also supports identifying best practices in diverse settings. CFIR consists of five key domains: (1) the implementation process, (2) innovation characteristics, (3) individual characteristics, (4) the inner setting, and (5) the outer setting.^25^, ^27^

### Data analysis

**Figure 1:**
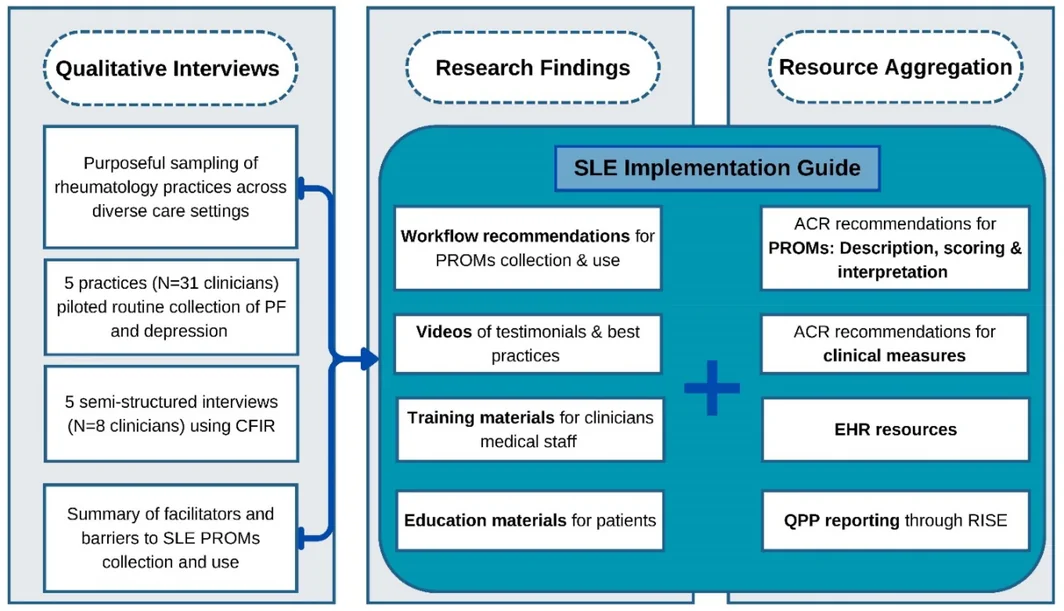
Development of the SLE Quality Measures Implementation Guide: slequalitymeasures.kotobee.com. ACR, American College of Rheumatology; CFIR, Consolidated Framework for Implementation Research; EHR, electronic health record; PF, physical function; PROM, patient‐reported outcome measure; QPP, Quality Payment Program; RISE, Rheumatology Informatics System for Effectiveness; SLE, systemic lupus erythematosus. Color figure can be viewed in the online issue, which is available at http://onlinelibrary.wiley.com/doi/10.1002/acr.80038/abstract.

We conducted a thematic analysis of open‐ended responses using both deductive and inductive techniques.^28^ Initially, an experienced qualitative researcher (CN) examined the data, applying deductive codes based on CFIR constructs to identify implementation barriers and facilitators while generating inductive codes to capture emerging ideas across interviews. These preliminary codes were then reviewed collaboratively with the research team, refined, and organized into a comprehensive codebook. Two coders (CW and CH) independently coded the transcripts; coding was reviewed to ensure consistency, and results were compiled into a matrix to identify key themes. Any discrepancies were resolved through discussion with a third coder (CN). Using an iterative consensus process,^29^ a qualitative researcher (CN) reviewed the matrix and generated themes and subthemes, supported by illustrative examples, which were then organized and mapped within the five CFIR domains: outer setting, inner setting, intervention characteristics, implementation process, and individual characteristics (Figure 1). Selected quotes were included to highlight important insights, such as innovations, resource availability, effective practices, and challenges related to collecting SLE outcome measures. The study adhered to the Consolidated Criteria for Reporting Qualitative Research guidelines (Supplementary Material 2).

### SLE Quality Measures Implementation Guide development

**Figure 2:**
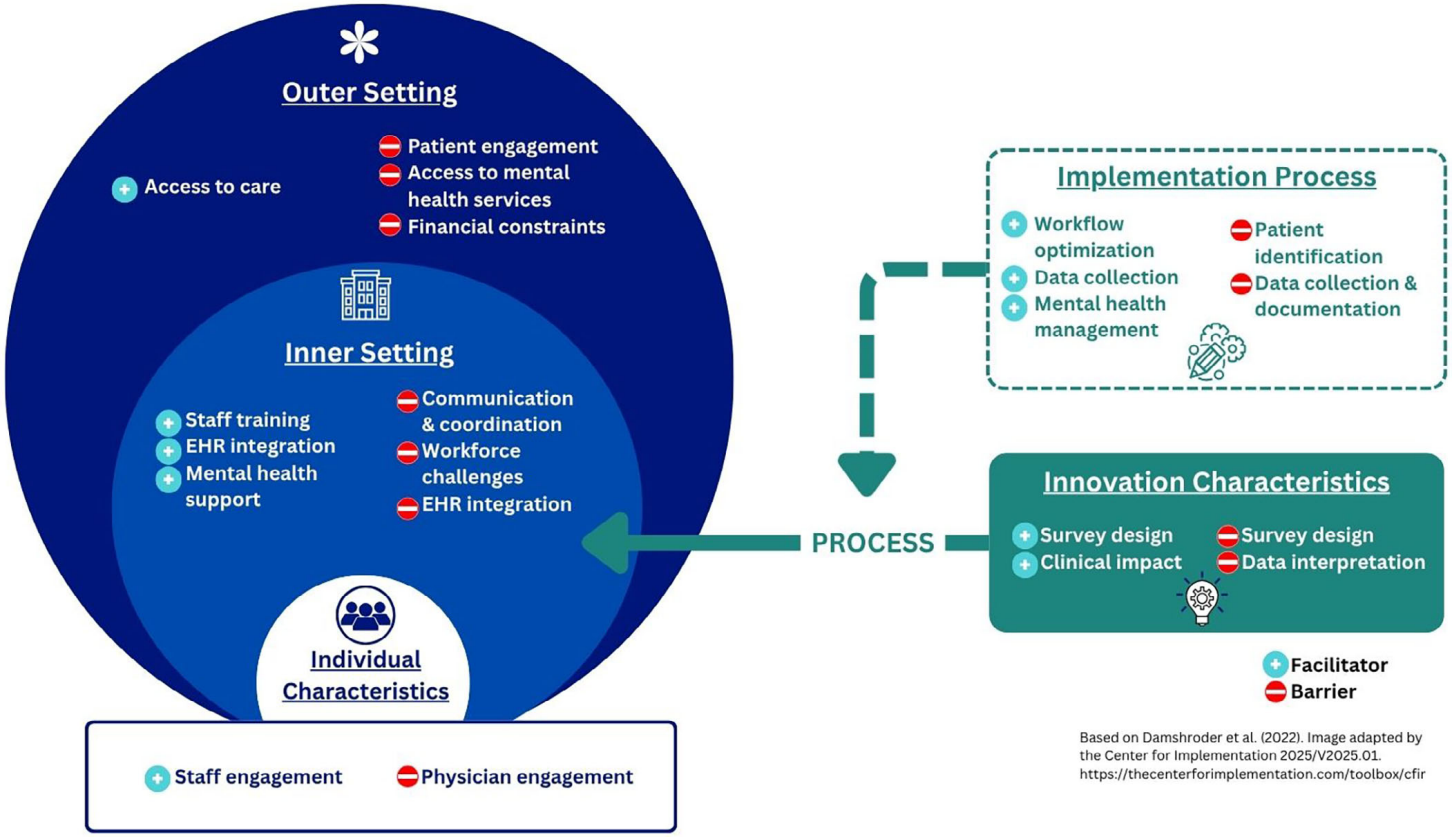
Generated themes as per the Consolidated Framework for Implementation Research domains. EHR, electronic health record.

Due to limited resources guiding rheumatologists on PROMs and how to routinely integrate them into clinical workflows, we used a consensus‐building approach based on iterative feedback cycles.^26^, ^30^, ^31^ This approach combined expert discussions, review of current literature, and feedback from semistructured interviews with trial practices that implemented ACR‐developed SLE PROMs, with funding support from the Centers for Disease Control and Prevention (CDC). Although these measures were published and approved by the ACR Board of Directors, they are not yet part of the Centers for Medicare and Medicaid Services’ Quality Payment Program (QPP). Insights from the practice interviews, combined with evidence‐based resources, informed the development of the SLE Quality Measures Implementation Guide: slequalitymeasures.kotobee.com (Figure 2), which provides practical tools to help rheumatology teams integrate PROMs into patient care. Best practices, challenges, and real‐world practice solutions were identified through interviews and incorporated into the implementation guide to ensure real‐world relevance. Consensus was operationalized as convergence among the expert team on the acceptability, usability, and feasibility of the guidance for routine clinical implementation. Educational and training materials for patients and staff were developed in response to concerns raised by interview participants and were pilot tested in practices to support more efficient and effective PROM collection and use with feedback gathered and participant suggestions incorporated iteratively. For context, impressions and testimonials from patients and rheumatologists who implemented SLE PROMs were included, highlighting their value and impact on care. In addition to PROMs, the implementation guide also emphasizes implementing clinical quality measures such as consistent hydroxychloroquine use, limiting glucocorticoid exposure, and regular lupus nephritis screening.^9^ The implementation guide was refined through iterative feedback from five rheumatologists and underwent user testing with team members and stakeholders, with edits made accordingly. Strategies were mapped using the Expert Recommendations for Implementing Change checklist to align with the evidence‐based SLE Quality Measures Implementation Guide (Supplementary Material 3).

## RESULTS

We conducted interviews with nine participants across the five RISE practices that participated in the pilot study, including five rheumatologists, two practice managers, one nurse practitioner, and one practice administrator. Practice characteristics are summarized in Table 1. Emerging themes and subthemes were organized into the five domains of the CFIR model.

**Table 1:** Characteristics of practices interviewed regarding SLE PROM collection*

Characteristic	Practice 1	Practice 2	Practice 3	Practice 4	Practice 5
Geographic division	South	South	South Atlantic	East South Central	South Atlantic
Practice type	Single specialty	Single specialty	Single specialty	Single specialty	Single specialty
Total clinicians at the practice, n	3	9	12	6	24
Clinicians engaged in the pilot, n	3	8	12	6	2
Patients with SLE diagnosis, n	711	566	703	999	883
Physical function					
Measure	RAPID‐3	MDHAQ	HAQ	MDHAQ	MDHAQ
Unique patients’ PF score collected per practice for patients with SLE	35	>150	65	>150	50
Unique patients’ PHQ‐8 responses concerning depression	0^a^	12	41	183	30

* All participating sites were Rheumatology Informatics System for Effectiveness–affiliated community practices that accept all insurance types. HAQ, Health Assessment Questionnaire; MDHAQ, Multidimensional Health Assessment Questionnaire; PHQ‐8, Patient Health Questionnaire 8; PROM, patient‐reported outcome measure; RAPID‐3, Routine Assessment of Patient Index Data 3; SLE, systemic lupus erythematosus.

^a^ Practice 1 returned 35 PHQ‐8 surveys that were scanned into the electronic health record and were not extractable by the medical record organization, so 0 unique responses were recorded.

### Implementation process

Practices described several strategies for integrating ACR‐recommended SLE PROMs into clinical workflows, which contributed to improved efficiency. Standardizing PROM collection at patient check‐in helped establish a foundational infrastructure for value‐based care (Table 2, quote 1). Structured processes, such as flagging patients with SLE or generating patient lists and embedding PROM forms in charts, enabled consistent identification of patients with SLE and incorporation of PROMs into patient records, streamlining their routine use (Table 2, quote 2). Some practices also implemented EHR alerts as a workflow adaptation to prompt staff to administer PROMs during each visit, further enhancing workflow efficiency (Table 2, quote 3). PF was already being collected by all practices, which facilitated implementation, whereas depression screening was not conducted at baseline. Routine collection of PROMs such as the PHQ‐8 at each visit reinforced their integration into clinical workflows (Table 2, quote 4). Most practices reported that using PROMs improved proactive symptom management, especially for mental health, with some initiating treatments such as antidepressants based on elevated PHQ‐8 scores (Table 2, quote 5). Despite these successes, several process‐related challenges affected consistent implementation. Some practices struggled to reliably identify patients with SLE ahead of visits, which disrupted PROM administration at check‐in (Table 3, quote 1). Staff occasionally missed administering PROMs when occupied with other tasks (Table 3, quote 2), and even when patients completed the forms, some scores were not entered into the EHR due to misplaced paperwork, staff oversight, or competing priorities (Table 3, quote 3).

**Table 2:** Current practices, benefits, and opportunities in SLE PROM collection: qualitative interview findings*

CFIR domain	Theme	Subtheme	Quotes
Implementation process	1. Workflow optimization	1. Standardizing PROMs collection at check‐in	Quote 1: “It's almost like laying down pipes and building plumbing for something to come. It's really helpful because that, what needs to be done in the community practice setting to get us ready for the world of value‐based medicine whenever that does hit.” (Practice 3)
		2. Flagging patients with SLE	Quote 2: “So [staff member] would run a report and then make a sheet for the front office manager, and she would put the papers in… It's part of the chart…” (Practice 3)
		3. Setting PROM reminder alerts	Quote 3: “We have an alert at the top, so if they have [a lupus diagnosis], it'll just say that they could put it in the alert section. So, when you click on it, you have to acknowledge it too, so that could be helpful…” (Practice 1)
	2. Data collection	1. Routine PROM collection	Quote 5: “My staff gives [the PHQ‐8] to the patients every time they come in.” (Practice 2)
	3. Mental health management	1. Managing symptoms	Quote 4: “We can always initiate them [patients] on antidepressant therapy in the clinic… and I think that usually makes a big difference.” (Practice 4)
Innovation characteristics	1. Survey design	1. Brief mental health questionnaires	Quote 7: “One of the easiest things to implement is the RAPID‐3. You get it on one piece of paper. Most patients could do it quickly.” (Practice 1)
	2. Clinical impact	1. Enhanced clinical decision‐making	Quote 8: “Having something like [the PHQ‐8] can be very helpful, because it also changes what your treatment options are, and what you're thinking about doing [treatment plan].” (Practice 1)
	3.	2. Patient‐centered care	Quote 9: “In chronic diseases, I think the emotional mental status should be sort of like the fifth vital sign… If you have a tool like [the PHQ‐8], just like we do CDAIs all the time…it can only take so little time and help patients in such a dramatic way.” (Practice 3)
		3. Strong patient–clinician rapport	Quote 10: “Even just in one diagnosis and being able to look at that [PRO forms] and the impacts and really see what's going on with your patients from a mental health perspective is so very important.” (Practice 5)
		4. Improved patient satisfaction and well‐being	Quote 11: “There was one patient that impressed me in how severely depressed she was… When we got her into counseling and medications…the PHQ‐8 went down to a zero from a 20. So that was really nice to see.” (Practice 4)
		5. Greater patient awareness of symptoms	Quote 12: “Patients would actually bring it up [their symptoms] and say, ‘Gosh, I didn't realize I was feeling this way or thinking these things until you asked the question.’” (Practice 3)
		6. Greater patient awareness of care process	Quote 13: “Once they put it on paper, they stare at it and I stare at it, we both have to address it… ‘I have a long‐standing history of depression, I didn't realize how much it is disabling, I need help.’” (Practice 2)
Individual characteristics	1. Staff engagement	1. Commitment and collaboration	Quote 14: “I credit [staff members] for recognizing that, ‘Hey, we're doing this.’ We need to identify the groups that do the different pieces of work, communicate with them, and then make it happen” (Practice 5)
Inner setting	1. Staff training	1. PROM trainings	Quote 15: “We have an onboarding process, so they're all trained to be able to do all of the things that every MA does… So, we do have an operating procedure for them.” (Practice 4)
	2.EHR integration	1. Electronic collection of measures	Quote 16: “So that [PROM form] is printed out for each patient and then that data is entered with that Magic Pen… they do that very fast so that by the time I go into the room, that data is already there.” (Practice 2)
		2. Automated score calculation	Quote 17: “There's a pre‐built [PROM tool in the EHR] where they just put in the scores, and the EMR automatically calculates it and gives us the score.” (Practice 4)
		3. Automated EHR data capture	Quote 18: “So, we have a company called Phreesia that does a pre‐visit for our basic data. They can enter it before they arrive in the office or when they first check‐in they get a little tablet. It's a multi‐page electronic [PROM] form that becomes part of their visit in their chart as informational data.” (Practice 3)
	3. Mental health support	1. Access to resources	Quote 19: “That actually is just a local website here [at the practice site] with combined resources that are available to everybody. It was available for all the patients that scored high [on PHQ‐8]. If it was over four.” (Practice 3)
Outer setting	1. Access to care	1. Proactive referrals for mental health	Quote 20: “You're making them [patients] recommendations that, ‘You need to go see your primary care, doctor, as soon as possible. You're not in a good situation,’ and then having them take that form or sending that over to the primary care provider is one option.” (Practice 1)

* CDAI, Clinical Disease Activity Index; CFIR, Consolidated Framework for Implementation Research; EHR, electronic health record; MA, medical assistant; PHQ‐8, Patient Health Questionnaire 8; PRO, patient‐reported outcome; PROM, PRO measure; RAPID‐3, Routine Assessment of Patient Index Data 3; SLE, systemic lupus erythematosus.

**Table 3:** Barriers to SLE PROM collection: qualitative interviews findings*

CFIR domain	Theme	Subtheme	Quotes
Implementation process	1. Patient identification	1. Gaps in identifying patients with SLE at check‐in	Quote 1: “The biggest issue we had was just identifying lupus patients prior to the visit.” (Practice 3)
	2. Data collection and documentation	1. Inconsistent collection of PROMs	Quote 2: “So, I think if they're [MAs] busy chatting with the patient, they're overlooking or forgetting to administer it.” (Practice 4)
		2. Missed documentation of scores in the EHR	Quote 3: “There's some data that got missing. I don't know where some of the paperwork went because… sometimes MAs are just forgetting to record [scores].” (Practice 4)
Innovation characteristics	1. Survey design	1. Lengthy PF questionnaire	Quote 3: “I mean, that's the big thing it's always a challenge of just the amount of time MDHAQ takes [to be filled out by patients].” (Practice 1)
		2. Sensitivity of mental health survey items	Quote 4: “For some people, it's triggering, they'll start crying when they're filling out the form [PHQ‐8].” (Practice 4)
	2. Interpretation of data	1. Physician's difficulty interpreting scores with comorbidities	Quote 5: “There's a high percentage of people that have fibromyalgia or other associated conditions… There's some other things going on.” (Practice 1)
		2. Patient difficulty interpreting scores and survey items	Quote 6: “The weakness in PROMs is the patient's own assessment, I find, is often inaccurate. Maybe we don't explain it enough. Sometimes I want to go back and ask if they understood the question [included in the PROM form] on a scale of 0 to 10, or if they're thinking 10 to 0.” (Practice 3)
Individual characteristics	1. Physician engagement	1. Inconsistent PF PROs discussion with patients	Quote 7: “[physician name], no he's lazy, he won't do any of it. He does it less than I do.” (Practice 1)
Inner setting	1. Communication and coordination	1. Communication gaps	Quote 15: “I did not make our providers totally aware that the questionnaires needed to be looked at [during visit].” (Practice 1)
	2. Workforce challenges	1. Staff turnover	Quote 16: “We just have a lot of staff turnover and so that introduces challenges at the beginning…when there's a new MA.” (Practice 4)
		2. Physician time constraints	Quote 17: “The biggest challenge really is the timeframe…we don't have much time…Getting all this done, especially if patients are doing it the day that they come in tends to be a little bit of an issue.” (Practice 1)
		3. Increased administrative burden	Quote 18: “Setting up the infrastructure within our office, having a system, generating patient flows, getting the staff involved, getting MAs involved, was the hard labor part.” (Practice 3)
	3. Integration into EHR	1. Unstructured fields	Quote 19: “The other challenging factor was that they had to build the [PROM] form in NextGen. And then where it's located in the chart is very difficult to find because it's not tracking over into our chart note.” (Practice 4)
		2. Manual data entry and scoring	Quote 20: “I had to manually do all of this [data entry]–follow the scores–because there's just no space. We can't see where the previous PHQ‐8 scores are in the EMR.” (Practice 4)
Outer setting	1. Patient engagement	1. Difficulty engaging older adults in mental health discussions	Quote 21: “The worst people are usually the elderly in regard to talking about mental health.” (Practice 1)
		2. Survey fatigue	Quote 22: “Some patients just say, ‘Well, I filled it [PROM form] out last time. Why do I have to do it again?’ We keep telling them, ‘You have to do it at each clinic visit. It tells us how you're doing. We can score it.’” (Practice 4)
		3. Discomfort with mental health topics	Quote 23: “These are things [mental health issues] that patients many times don't voluntarily share and they might not volunteer it because they're not comfortable.” (Practice 3)
	1. Access to mental health services	1. Long wait time for mental health services	Quote 24: “It's just the timing of referrals to mental health provider. It usually takes a very long time…” (Practice 1)
	2. Financial constraints	1. High costs of physical therapy	Quote 25: “I think patients are not resistant, but… it's [cost of physical therapy] expensive…the co‐pay costs add up, and that's a big hurdle for some of our patients.” (Practice 3)
		2. Limited insurance coverage	Quote 26: “I think it is the fact that a lot of insurers don't cover mental health… That becomes the issue.” (Practice 2)

* CFIR, Consolidated Framework for Implementation Research; EHR, electronic health record; EMR, electronic medical record; MA, medical assistant; MDHAQ, Multidimensional Health Assessment Questionnaire; PHQ‐8, Patient Health Questionnaire 8; PROM, patient‐reported outcome measure; SLE, systemic lupus erythematosus.

### Innovation characteristics

Overall, practices highlighted the value of ACR‐recommended PROMs in SLE care as both feasible and impactful tools. Survey design was considered practical, with brief instruments such as the RAPID‐3 noted as easy for patients to complete quickly and consistently (Table 3, quote 7). All practices highlighted the positive clinical impact of PROM collection on patient care. PROMs enhanced clinical decision‐making by uncovering important mental health concerns that informed treatment planning, an unexpected but highly valued benefit (Table 3, quote 8). PROMs promoted a patient‐centered approach by recognizing emotional well‐being as key to managing chronic disease (Table 2, quote 9). They also enhanced patient–provider communication, fostering discussions about mental health and enabling timely referrals (Table 2, quote 10). Ultimately, clinicians perceived that PROM‐guided SLE care improved patient satisfaction and well‐being, particularly through better mental health outcomes (Table 2, quote 11). On the patient side, as reported by clinicians, all practices noted that PROMs enhanced self‐awareness and engagement. Clinicians noted that using structured questions often uncovered previously unrecognized issues, prompting deeper conversations about symptoms (Table 2, quote 12) and that articulating experiences through PROMs helped patients better understand their care and seek support (Table 2, quote 13).

Despite these benefits, several challenges limited the ease of PROM implementation. Survey design was a key barrier, with practices using the MDHAQ to assess patient PF, describing it as lengthy and disruptive to workflow (Table 3, quote 3). One practice noted that emotionally sensitive PHQ‐8 items occasionally triggered strong patient reactions, requiring immediate follow‐up and clinical support (Table 3, quote 4). Interpreting PROM data also proved difficult in some cases. A few practices reported challenges interpreting PF scores in patients with comorbid conditions like fibromyalgia, in which overlapping symptoms obscured the source of dysfunction (Table 3, quote 5). Others observed that patients misunderstood certain questions or response scales, leading to inaccurate responses (Table 3, quote 6).

### Individual characteristics

Individual‐level factors, including physician and medical support staff engagement, played an important role in successfully integrating ACR‐recommended PROMs into SLE care. Two practices reported that staff commitment, collaboration, and communication enabled effective workflow implementation and sustained use (Table 2, quote 14). In contrast, in one case, PF measures were deprioritized by clinicians, given the shift of focus, resulting in inconsistent use and discussion with patients (Table 2, quote 7).

### Inner setting

Supportive inner setting factors, such as staff training, technological support, and access to in‐house mental health support, facilitated the integration of SLE PROMs into routine care. All practices implemented structured onboarding and training to ensure staff could administer and process PROMs consistently (Table 2, quote 15). In addition, technological support via EHR integration played a crucial role in streamlining PROM workflows. Electronic collection of measures using digital tools, such as iPads and “Magic Pens,” enabled staff to input and review responses before the clinician saw the patient, ensuring timely access to data (Table 2, quote 16).

Some practices implemented automatic score calculation, which reduced provider workload and ensured the immediate availability of scores (Table 2, quote 17). One practice used external platforms such as Phreesia to collect PROMs electronically before visits, automatically uploading data into medical records to improve documentation and track patient progress over time (Table 2, quote 18). A few practices also provided mental health resources to connect patients to care, such as printed lists of local services for those with high PHQ‐8 scores (Table 3, quote 19).

However, some practices reported communication gaps between providers and staff, which occasionally hindered consistent review and use of questionnaire data (Table 3, quote 15). Workforce challenges such as high staff turnover, physician time constraints, and increased administrative burden also complicated implementation. Ongoing turnover created repeated training demands to maintain workflow consistency (Table 3, quote 16), whereas time constraints during appointments made it difficult to review PROMs, especially when completed on the visit, leading to delays (Table 3, quote 17). Building the necessary PROM infrastructure also required significant administrative effort to coordinate workflows (Table 3, quote 3). EHR integration posed widespread technical and logistical barriers for nearly all practices. PROM forms were often stored in unstructured locations, making them difficult to access during clinical encounters or integrate into progress notes (Table 3, quote 19). Furthermore, the absence of automated data entry and scoring required manual tracking, which increased staff workload and reduced overall efficiency (Table 3, quote 20).

### Outer setting

Regarding external factors, all practices emphasized the value of collecting SLE PROMs, particularly PHQ‐8, to facilitate proactive referrals and access to mental health services for patients scoring above the threshold (Table 2, quote 20). However, patient engagement also presented challenges, particularly around mental health. One practice reported difficulty engaging older adults in discussions of emotional well‐being, noting generational reluctance to disclose mental health concerns (Table 3, quote 21). Survey fatigue was another common issue reported by most of the practices because many patients questioned the need to complete PROMs on every visit despite staff explanations (Table 3, quote 22). Discomfort discussing mental health also emerged as a barrier, with some patients unwilling to share emotional concerns due to stigma or discomfort (Table 3, quote 23). Additionally, most practices reported long wait times for mental health providers, often delaying timely access to specialist care. To address this, many patients initially received mental health support from their primary care physicians while awaiting appointments with specialists such as psychiatrists (Table 3, quote 24). Financial constraints further hindered care: the high cost of physical therapy, including accumulating copays, discouraged some patients from pursuing recommended services (Table 3, quote 25), and limited insurance coverage for mental health care led to substantial out‐of‐pocket expenses, causing some patients to delay or forgo treatment despite clinical need (Table 3, quote 26).

### SLE Quality Measures Implementation Guide development

Results from semistructured interviews with five pilot practices helped identify key challenges and opportunities to integrate PROMs into routine SLE clinical care. Insights from these interviews, combined with ACR guidelines and other evidence‐based resources, informed the development of the SLE Quality Measures Implementation Guide (slequalitymeasures.kotobee.com). The SLE Quality Measures Implementation Guide, launched on the ACR website in May 2025, is an online resource designed to support clinicians and staff in implementing SLE quality measures to deliver high‐quality, patient‐centered care. It includes detailed guidance on using PROMs recommended by the ACR for SLE along with downloadable forms, scoring instructions, workflows, and explanations to aid understanding.

Reflecting themes from the interviews, the implementation guide also features videos on best practices, patient education materials, staff training resources, and validated translations of PROMs in Spanish. To address common barriers, it includes translated materials in Spanish and offers considerations for collecting PROMs in diverse populations. Additional resources include a downloadable staff training guide to support effective PROM collection and a patient education guide to improve awareness of their importance in SLE care. To further support clinical workflows, the implementation guide also offers tips for succeeding in the QPP.

## DISCUSSION

Addressing PROMs in SLE is important to help develop a comprehensive care plans aligned with patients’ priorities and values. This study identifies key real‐world barriers and facilitators to implementing PROMs in routine SLE care and translates these insights into practical strategies within the ACR SLE Quality Measures Implementation Guide. By applying the CFIR framework across five diverse clinical practices, we developed workflows and digital PROM templates, shaped by clinician beliefs, clinical workflows, organizational readiness, and the availability of digital tools, within the ACR SLE Quality Measures Implementation Guide to support adoption across diverse practices. This guide is the first to enable comprehensive care delivery focused on both clinical and patient‐reported outcomes as part of routine SLE care.

Individuals with SLE often have worse‐than‐average scores on PROMs, resulting in subsequent morbidity, disability, and mortality.^30^ Therefore, PROMs complement clinical measures to support comprehensive care planning and align treatment plans with patient priorities and values in SLE.^32^ Although most research has focused on validating these measures and applying them in clinical trials,^33^, ^34^ there is still a notable gap in practical guidance on how to effectively integrate PROMs into routine clinical workflows, particularly across different practices.^33^, ^35^, ^36^, ^37^ Limited resources to help collect, interpret, and apply PROM data within existing workflows across practices are a significant barrier; however, additional unaddressed barriers, such as time constraints, insufficient training, and uncertainty about the clinical utility of these measures, further limit widespread adoption.^33^, ^35^, ^38^ To directly address this gap, we pilot tested the implementation of ACR‐recommended PROMs for SLE^9^, ^39^ across five diverse practices to understand barriers, detractors, and motivators for adopting PROMs as part of routine workflows.

Leveraging the CFIR framework, we identified key barriers and motivators to inform the ACR SLE Quality Measures Implementation Guide. More importantly, CFIR helped us understand how individual beliefs, clinical workflows, and organizational factors affect the collection and use of SLE PROMs. For example, standardizing check‐in procedures and implementing EHR alerts facilitated consistent PROM collection and patient identification. Accordingly, the guide includes a section defining methods for identifying eligible patient lists, either digitally or manually, and provides customizable swim lane diagrams to support workflow implementation across diverse pratice settings.

Staff onboarding and training were also reported as essential for sustained PROM use. The guide includes a dedicated medical assistant training module and a “Testimonials and Advice from Rheumatologists and Patients” section that showcases real‐world strategies from pilot sites. These include EHR alerts for clinical staff, digital patient identification workflows, and the use of electronic tools to streamline PROM completion during routine SLE visits. Unlike prior studies that focus on PROM psychometrics or trial‐based applications, this study explores how PROMs can be embedded in routine workflows by leveraging technology (eg, EHR alerts, and automatic scoring) and staff engagement. The implementation guide incorporates these insights by providing training guides, workflow templates, and culturally adapted materials to support implementation across varied settings.

Poor mental health and PF are associated with poor SLE outcomes. Therefore, assessment of mental health and PF using tools like the PHQ‐8 or Patient‐Reported Outcomes Measurement Information System (PROMIS)– Short Form Depression and the MDHAQ or PROMIS Physical Function, respectively, were included as recommended PROMs for SLE visits.^3^, ^9^, ^19^ Particularly for the mental health assessment during pilot testing, clinicians reported that using PROMs like the PHQ‐8 enabled early identification of depression and more timely intervention. These findings were consistent with prior studies showing that PROMs, including mental health screening, support clinical decision‐making and enhance patient–provider communication.^7^, ^21^ The ACR SLE guide curates validated resources, including links to national and regional mental health resources and culturally adapted materials in multiple languages. PF assessments were easier to implement in practices already using instruments such as the MDHAQ for all patients, facilitating higher adoption. However, uncertainty in interpreting PF scores among patients with comorbidities and the length of some questionnaires remained challenges. To address these issues, the SLE Quality Measures Implementation Guide provides recommendations for asynchronous PROM completion (before or between clinic touchpoints). In addition, a section on reimbursement programs, including the Quality Payment Program (QPP) has been incorporated to incentivize practices to measure both PROMs and clinical SLE measures, aiming toward improving overall SLE care quality. The development of a publicly accessible, multimedia‐rich, and interactive SLE Quality Measures Implementation Guide tailored to diverse practice settings represents a novel approach to promoting the use of PROMs and clinical SLE measure adoption across clinics.

Despite promising outcomes, several limitations emerged. First, practices faced challenges in reliably identifying patients with SLE, maintaining consistent PROM administration, and interpreting scores in patients with comorbid conditions. Second, survey fatigue and the emotional sensitivity of items (eg, the PHQ‐8) occasionally hindered patient engagement. Third, technical barriers, such as unstructured EHR fields and manual data entry, increased staff burden. In addition, the tools were developed and tested only within a single EHR system used by the five pilot practices. External factors, such as limited insurance coverage and long wait times for mental health services, also constrained the impact of PROM‐guided care. Lastly, implementation outcome metrics to evaluate the adoption of PROM and clinical measures data collection during SLE visits after launching the SLE Quality Measures Implementation Guide have yet to be assessed.

In conclusion, the ACR SLE Quality Measures Implementation Guide translates real‐world insights into actionable strategies for embedding PROMs and longitudinal clinical measures into routine care. It supports scalable, patient‐centered SLE management through technology, staff engagement and training, and structured workflows. Finally, the digital SLE guide provides unique yet customizable strategies to embed digital quality measure assessments, particularly PROMs, in routine clinical care to ensure better care delivery standards and improve care quality in SLE.

## AUTHOR CONTRIBUTIONS

All authors contributed to at least one of the following manuscript preparation roles: conceptualization AND/OR methodology, software, investigation, formal analysis, data curation, visualization, and validation AND drafting or reviewing/editing the final draft. As corresponding author, Dr Garg confirms that all authors have provided the final approval of the version to be published, and takes responsibility for the affirmations regarding article submission (eg, not under consideration by another journal), the integrity of the data presented, and the statements regarding compliance with institutional review board/Declaration of Helsinki requirements. All authors contributed to the investigation, formal analysis of the data, and writing, reviewing, and editing of the manuscript.

## Supporting information


**Disclosure form**.

Supplementary Material 1:

Supplementary Material 2:

Supplementary Material 3:
