# Supplementary material for: Integrating Patient‐Reported Quality Measures in Systemic Lupus Erythematosus: Development of the American College of Rheumatology Implementation Guide
Source: Arthritis Care Res (Hoboken). 2026 Apr 29;78(10):1467–77. doi: 10.1002/acr.80038 (PMC13613259; doi:10.1002/acr.80038)
Supplement: Supplementary file 3 [file ACR-78-1467-s001.docx]

**ERIC Strategy	ERIC Definition (updated with interested parties/stakeholders)**

| Did this project employ any of these ERIC **infrastructure change strategies** to promote uptake of an evidence-based practice?  Please select all that apply or select "None of the Above." | ☐ | Change physical structure and equipment | Evaluate current configurations and adapt, as needed, the physical structure and/or equipment (e.g. , changing the layout of a room, adding equipment) to best accommodate the targeted innovation |
| --- | --- | --- | --- |
|  | ☐ | Change record systems | Change records systems to allow better assessment of implementation or clinical outcomes |
|  | ☐ | Change service sites | Change the location of clinical service sites to increase access |
|  | ☐ | Start a dissemination organization | Identify or start a separate organization that is responsible for disseminating the clinical innovation. It could be a for-profit or non-profit organization |
|  | ☐ | Mandate change | Have leadership declare the priority of the innovation and their determination to have it implemented |
|  | ☐ | Create or change credentialing and/or licensure standards | Create an organization that certifies clinicians in the innovation or encourage an existing organization to do so. Change governmental professional certification or licensure requirements to include delivering the innovation. Work to alter continuing education requirements to shape professional practice toward the innovation |
|  |  |  |  |
|  | ☐ | Change liability laws | Participate in liability reform efforts that make clinicians more willing to deliver the clinical innovation |
|  | ☐ | Change accreditation or membership requirements | Strive to alter accreditation standards so that they require or encourage use of the clinical innovation. Work to alter membership organization requirements so that those who want to affiliate with the organization are encouraged or required to use the clinical innovation |
|  | ☐ | Other infrastructure change strategies (please specify): |  |
|  | ✓ | None of the above |  |

| Did this project employ any of these ERIC **financial strategies** to promote uptake of an evidence-  based practice? Please select all that apply or select "None of the  Above." | ☐ | Access new funding | Access new or existing money to facilitate the implementation |
| --- | --- | --- | --- |
|  | ☐ | Alter incentive/allowance structures | Work to incentivize the adoption and implementation of the clinical innovation |
|  | ☐ | Develop disincentives | Provide financial disincentives for failure to implement or use the clinical innovations |
|  | ☐ | Fund and contract for the clinical innovation | Governments and other payers of services issue requests for proposals to deliver the innovation, use contracting processes to motivate providers to deliver the clinical innovation, and develop new funding formulas that make it more likely that providers will deliver the innovation |
|  | ☐ | Make billing easier | Make it easier to bill for the clinical innovation |
|  | ☐ | Place innovation on fee for service lists/formularies | Work to place the clinical innovation on lists of actions for which providers can be reimbursed (e.g. , a drug is placed on a formulary, a procedure is now reimbursable) |
|  | ☐ | Alter patient/consumer fees | Create fee structures where patients/consumers pay less for preferred treatments (the clinical innovation) and more for less-preferred treatments |
|  | ☐ | Use capitated payments | Pay providers or care systems a set amount per patient/consumer for delivering clinical care |
|  | ☐ | Use other payment schemes | Introduce payment approaches (in a catch-all category) |
|  | ☐ | Create new clinical teams | Change who serves on the clinical team, adding different disciplines and different skills to make it more likely that the clinical innovation is delivered (or is more successfully delivered) |
|  | ☐ | Facilitate relay of clinical data to providers | Provide as close to real-time data as possible about key measures of process/outcomes using integrated modes/channels of communication in a way that promotes use of the targeted innovation |
|  | ☐ | Revise professional roles | Shift and revise roles among professionals who provide care, and redesign job characteristics |
|  | ✓ | Remind clinicians | Develop reminder systems designed to help clinicians to recall information and/or prompt them to use the clinical innovation |
|  | ✓ | Develop resource sharing agreements | Develop partnerships with organizations that have resources needed to implement the innovation |
|  | ☐ | Other financial strategies (please specify): |  |
|  | ☐ | None of the above |  |
| Did this project employ any of these ERIC **interactive assistance strategies** to promote uptake of an evidence-based practice?  Please select all that apply or select "None of the Above." | ☐ | Facilitation | A process of interactive problem solving and support that occurs in a context of a recognized need for improvement and a supportive interpersonal relationship |
|  | ☐ | Provide local technical assistance | Develop and use a system to deliver technical assistance focused on implementation issues using local personnel |
|  | ☐ | Provide clinical supervision | Provide clinicians with ongoing supervision focusing on the innovation. Provide training for clinical supervisors who will supervise clinicians who provide the innovation |
|  | ✓ | Centralize technical assistance | Develop and use a centralized system to deliver technical assistance focused on implementation issues |
|  | ✓ | Other provide interactive assistance strategies (please specify): | Using the RISE registry to track performance (Chapter 6 of the guide), EHR resources for SLE care (Chapter 7 of the toolkit) and tips for succeeding in Quality Payment Program (QPP) (Chapter 8 of the guide). |
|  | ☐ | None of the above |  |

| Did this project employ any of these ERIC **care tailoring strategies** to promote uptake of an evidence- based practice? Please select all that apply or select "None of the  Above." | ✓ | Use data experts | Involve, hire, and/or consult experts to inform management on the use of data generated by implementation efforts |
| --- | --- | --- | --- |
|  | ☐ | Use data warehousing techniques | Integrate clinical records across facilities and organizations to facilitate implementation across systems |
|  | ✓ | Tailor strategies | Tailor the implementation strategies to address barriers and leverage facilitators that were identified through earlier data collection |
|  | ✓ | Promote adaptability | Identify the ways a clinical innovation can be tailored to meet local needs and clarify which elements of the innovation must be maintained to preserve fidelity |
|  | ☐ | Other tailor strategies (please specify): |  |
|  | ☐ | None of the above |  |

| Did this project employ any of these ERIC **training or educational strategies** to promote uptake of an evidence-based practice?  Please select all that apply or select "None of the Above." | ✓ | Conduct educational meetings | Hold meetings targeted toward different interested party/stakeholder groups (e.g. , providers, administrators, other organizational interested parties/stakeholders, and community, patient/consumer, and family interested parties/stakeholders) to teach them about the clinical innovation |
| --- | --- | --- | --- |
|  | ☐ | Conduct educational outreach visits | Have a trained person meet with providers in their practice settings to educate providers about the clinical innovation with the intent of changing the provider’s practice |
|  | ✓ | Conduct ongoing training | Plan for and conduct training in the clinical innovation in an ongoing way |
|  | ✓ | Create a learning collaborative | Facilitate the formation of groups of providers or provider organizations and foster a collaborative learning environment to improve implementation of the clinical innovation |
|  | ✓ | Develop educational materials | Develop and format manuals, toolkits, and other supporting materials in ways that make it easier for interested parties/stakeholders to learn about the innovation and for clinicians to learn how to deliver the clinical innovation |
|  | ✓ | Distribute educational materials | Distribute educational materials (including guidelines, manuals, and toolkits) in person, by mail, and/or electronically |
|  | ✓ | Provide ongoing consultation | Provide ongoing consultation with one or more experts in the strategies used to support implementing the innovation |
|  | ✓ | Use train-the-trainer strategies | Train designated clinicians or organizations to train others in the clinical innovation |
|  | ☐ | Make training dynamic | Vary the information delivery methods to cater to different learning styles and work contexts, and shape the training in the innovation to be interactive |
|  | ☐ | Shadow other experts | Provide ways for key individuals to directly observe experienced people engage with or use the targeted practice change/innovation |
|  | ☐ | Work with educational institutions | Encourage educational institutions to train clinicians in the innovation |
|  | ☐ | Other train or educate strategies (please specify): |  |
|  | ☐ | None of the above |  |

| Did this project employ any of these ERIC **interested**  **party/stakeholder development strategies** to promote uptake of an evidence-based practice?  Please select all that apply or select "None of the Above." | ☐ | Build a coalition | Recruit and cultivate relationships with partners in the implementation effort |
| --- | --- | --- | --- |
|  | ☐ | Conduct local consensus discussions | Include local providers and other interested parties/stakeholders in discussions that address whether the chosen problem is important and whether the clinical innovation to address it is appropriate |
|  | ☐ | Obtain formal commitments | Obtain written commitments from key partners that state what they will do to implement the innovation |
|  | ☐ | Recruit, designate, and train for leadership | Recruit, designate, and train leaders for the change effort |
|  | ☐ | Inform local opinion leaders | Inform providers identified by colleagues as opinion leaders or “educationally influential” about the clinical innovation in the hopes that they will influence colleagues to adopt it |
|  | ✓ | Capture and share local knowledge | Capture local knowledge from implementation sites on how implementers and clinicians made something work in their setting and then share it with other sites |
|  | ☐ | Identify and prepare champions | Identify and prepare individuals who dedicate themselves to supporting, marketing, and driving through an implementation, overcoming indifference or resistance that the intervention may provoke in an organization |
|  | ☐ | Organize clinician implementation team meetings | Develop and support teams of clinicians who are implementing the innovation and give them protected time to reflect on the implementation effort, share lessons learned, and support one another’s learning |
|  | ☐ | Use advisory boards and workgroups | Create and engage a formal group of multiple kinds of interested parties/stakeholders to provide input and advice on implementation efforts and to elicit recommendations for improvements |
|  | ✓ | Use an implementation advisor | Seek guidance from experts in implementation |
|  | ✓ | Promote network weaving | Identify and build on existing high-quality working relationships and networks within and outside the organization, organizational units, teams, etc. to promote information sharing, collaborative problem-solving, and a shared vision/goal related to implementing the innovation |
|  | ☐ | Model and simulate change | Model or simulate the change that will be implemented prior to implementation |
|  | ✓ | Develop academic partnerships | Partner with a university or academic unit for the purposes of shared training and bringing research skills to an implementation project |
|  | ✓ | Identify early adopters | Identify early adopters at the local site to learn from their experiences with the practice innovation |
|  | ✓ | Visit other sites | Visit sites where a similar implementation effort has been considered successful |
|  | ☐ | Develop an implementation glossary | Develop and distribute a list of terms describing the innovation, implementation, and interested parties/stakeholders in the organizational change |
|  | ☐ | Involve executive boards | Involve existing governing structures (e.g. , boards of directors, medical staff boards of governance) in the implementation effort, including the review of data on implementation processes |
|  | ☐ | Other interested parties/stakeholder development strategies (please specify): |  |
|  | ☐ | None of the above |  |
| Did this project employ any of these ERIC **evaluative and**  **iterative strategies** to promote uptake of an evidence-based  practice? Please select all that  apply or select "None of the  Above." | ✓ | Assess for readiness and identify barriers and facilitators | Assess various aspects of an organization to determine its degree of readiness to implement, barriers that may impede implementation, and strengths that can be used in the implementation effort |
|  | ☐ | Conduct local needs assessment | Collect and analyze data related to the need for the innovation |
|  | ☐ | Develop a formal implementation blueprint | Develop a formal implementation blueprint that includes all goals and strategies.  The blueprint should include the following: 1) aim/purpose of the implementation; 2) scope of the change (e.g. , what organizational units are affected); 3) timeframe and milestones; and 4) appropriate performance/progress measures. Use and update this plan to guide the implementation effort over time |
|  | ☐ | Stage implementation scale up | Phase implementation efforts by starting with small pilots or demonstration projects and gradually move to a system wide rollout |
|  | ☐ | Conduct cyclical small tests of change | Implement changes in a cyclical fashion using small tests of change before taking changes system-wide. Tests of change benefit from systematic measurement, and results of the tests of change are studied for insights on how to do better. This process continues serially over time, and refinement is added with each cycle |
|  | ✓ | Audit and provide feedback | Collect and summarize clinical performance data over a specified time period and give it to clinicians and administrators to monitor, evaluate, and modify provider behavior |
|  | ✓ | Develop and implement tools for quality monitoring | Develop, test, and introduce into quality-monitoring systems the right input—the appropriate language, protocols, algorithms, standards, and measures (of processes, patient/consumer outcomes, and implementation outcomes) that are often specific to the innovation being implemented |
|  | ✓ | Develop and organize quality monitoring systems | Develop and organize systems and procedures that monitor clinical processes and/or outcomes for the purpose of quality assurance and improvement |
|  | ☐ | Purposely reexamine the implementation | Monitor progress and adjust clinical practices and implementation strategies to continuously improve the quality of care |
|  | ☐ | Obtain and use  patients/consumers and family feedback | Develop strategies to increase patient/consumer and family feedback on the implementation effort |
|  | ☐ | Other evaluative and iterative strategies (please specify): |  |
|  | ☐ | None of the above |  |

| Did this project employ any of these ERIC **strategies to engage patient consumers** to promote uptake of an evidence-based  practice? Please select all that  apply or select "None of the  Above." | ☐ | Involve patients/consumers and family members | Engage or include patients/consumers and families in the implementation effort |
| --- | --- | --- | --- |
|  | ☐ | Prepare patients/consumers to be active participants | Prepare patients/consumers to be active in their care, to ask questions, and specifically to inquire about care guidelines, the evidence behind clinical decisions, or about available evidence-supported treatments |
|  | ☐ | Intervene with patients/consumers to enhance uptake and adherence | Develop strategies with patients to encourage and problem solve around adherence |
|  | ☐ | Use mass media | Use media to reach large numbers of people to spread the word about the clinical innovation |
|  | ☐ | Increase demand | Attempt to influence the market for the clinical innovation to increase competition intensity and to increase the maturity of the market for the clinical innovation |
|  | ☐ | Other engagement strategies (please specify): |  |
|  | ✓ | None of the above |  |
