# Supplementary material for: Integrating Patient‐Reported Quality Measures in Systemic Lupus Erythematosus: Development of the American College of Rheumatology Implementation Guide
Source: Arthritis Care Res (Hoboken). 2026 Apr 29;78(10):1467–77. doi: 10.1002/acr.80038 (PMC13613259; doi:10.1002/acr.80038)
Supplement: Supplementary file 4 [file ACR-78-1467-s003.docx]

**Interview Guide**

**Physical Function**

1. Please describe the current workflow in your practice for collecting physical function PROs.
   a. Which staff members are involved in this workflow?
   b. What are the key elements of this workflow?

i. What is the time involved in this step

c. What type of barriers or challenges did you face when collecting physical function? (EHR, Staff, patients)
d. How did you manage these challenges?

2. How is the data recorded/uploaded into the EHR?
   a. Where in the EHR is this data being recorded? (structured fields or notes).
   b. How did this pilot lead to changes in how you document physical function results in the EHR?
   c. Were the ACR templates/custom forms helpful for capturing data?

i. Did you create a custom section/feature to record results?

3. Can you describe how physical function screening results are used (if at all) by rheumatologists or other staff in your practice?
   a. How are results discussed with the patient?
   b. Who do you share the results with?
   c. What is the action plan if the patient has identified physical function concerns?
   d. Any positive stories you would like to share?

4. What type of changes did you notice in your practice after incorporating physical function screening?

5. What are your future goals in terms of collecting physical function?
   a. Which PF tool do you plan to use? Why?

**Depression**

1. Please describe the current workflow in your practice for collecting depression PROs.
   a. Which staff members are involved in this workflow?
   b. What are the key elements of this workflow?

i. What is the time involved in this step

2. What type of barriers or challenges did you face when collecting depression? (EHR, Staff, patients)
   a. How did you manage these challenges?

3. How is the data entered into the EHR?
   b. Where in the EHR is this data being recorded? (structured fields or notes).
   c. How did this pilot lead to changes in how you document depression report in the EHR?
   d. Were the ACR templates/custom forms helpful for capturing data?

i. Did you create a custom section/feature to record results?

4. Please describe how the results of the depression screening are used (if at all) by rheumatologists or other staff in your practice?
   e. How are results discussed with the patient?
   f. Who do you share the results with?
   g. What is the action plan if the patient has a depression diagnosis?
   h. Probe – Any positive stories you would like to share?

5. What type of changes did you notice in your practice after incorporating depression screening?

6. What are your future goals in terms of collecting depression measures?
   a. Which depression measures do you plan to use? Why?

**Global Questions**

1. Can you share any feedback from patients who participated in the pilot?
2. Any patients opt-out of participating? Why?

3. What were some of the best aspects of participating in this pilot?

4. What were some of the biggest challenges of participating in this pilot?

5. How could the ACR address barriers in collecting PROs from people with SLE over the long term?
   a. Probe: Language barriers/translation services

6. Did you use the ACR-created NextGen templates (the Zip file that was a NextGen specific template for your clinical notes)?
